# Porous Structure of Ultra-High-Performance Fibre-Reinforced Concretes

**DOI:** 10.3390/ma14071637

**Published:** 2021-03-26

**Authors:** Manuel Valcuende, Josep R. Lliso-Ferrando, Marta Roig-Flores, José M. Gandía-Romero

**Affiliations:** 1 Department of Architectural Constructions, School of Architecture, Universitat Politècnica de València, Camino de Vera, s/n., 46022 Valencia, Spain; 2 Research Institute for Molecular Recognition and Technological Development (IDM), Universitat Politècnica de València, Camino de Vera, s/n., 46022 Valencia, Spain; jollife2@alumni.upv.es (J.R.L.-F.); joganro@csa.upv.es (J.M.G.-R.); 3 Concrete Science and Technology University Institute (ICITECH), Universitat Politècnica de València, Camino de vera s/n, 46022 Valencia, Spain; roigma@uji.es

**Keywords:** ultra-high-performance fibre-reinforced concrete, porosity, water porosity, oxygen permeability

## Abstract

The aim of this experimental work was to study the porous structure of Ultra-High-Performance Fibre-Reinforced Concretes (UH) made with different fibre volume contents (0%, 1%, 2%) under several curing conditions (laboratory environment, 20 °C, 60 °C, 90 °C), comparing the results with those recorded for ordinary, high strength and very high strength concretes. Scanning electron microscopy, mercury intrusion porosimetry, thermogravimetry, water absorption and oxygen permeability tests were carried out. The results showed a low portlandite content in UH (in the order of 75% lower than in concrete C50) and a low degree of hydration, but they rise with curing temperature. These concretes have a very fine porous structure, with a high concentration of pores on the nanoscale level, below 0.05 µm. Their porosity accessible to water is consequently around 7-fold lower than in conventional (C30), 6-fold lower than in high-strength (C50) and 4-fold lower than in very high-strength (C90) concretes. Their oxygen permeability is at least one order of magnitude lower than in C90, two orders of magnitude lower than in C50 and three orders of magnitude lower than in C30. The percentage of added steel fibre does not affect the UH porous structure.

## 1. Introduction

Efficient resource management is one of the goals pursued by today’s society. Enhancing the sustainability and streamlining of using construction materials entails prolonging structures’ service life. Ultra-high performance fibre reinforced concrete (UHPFRC) may be an alternative for some structure types, especially in very aggressive environments, because it draws from the latest concrete industry developments to deliver high strength and durability. Although such concretes require large amounts of cement, according to some studies [1] they are not necessarily more costly or more harmful to the environment than conventional concrete because the bearing cross section required for a given load is smaller, but especially because their service life may be 2-fold, or longer. In fact, further to a study published by the Quebec Ministry of Transport, the cost of a bridge built with 50 MPa to 60 MPa concrete was 8% lower than one built with 35 MPa concrete [2].

The French design manual “Bétons fibres à ultra-hautes performances” [3] defines ultra-high-performance concretes as those with a compressive strength of 150 MPa or higher, while other standards, such as the Swiss guide [4] or ASTM C1856, set the lower limit at 120 MPa. Pierre Richard began to develop what were initially called Reactive Powder Concretes [5] in the 1990s. De Larrard was the first to later coin the term ultra-high-performance concrete for this the new material [6], first applied in civil engineering for a pedestrian bridge in Sherbrooke, Canada [7]. It has since been used at other sites, such as the nuclear power plants at Cattenom and Civaux in France [8] and offshore marine platforms [9].

UHPFRC is made with very low water/binder (w/b) ratios, no coarse aggregate and very high cement, superplasticizer and additional (especially silica fume and ground quartz) contents [10,11,12] to produce a very homogeneous material with high mechanical strength and a dense microstructure [13,14,15]. In many dosages, the microsilica content is equal to or higher than 20% (by cement wt%), even though Khosravani et al. [16] obtained a better mechanical behaviour with a 10%. Furthermore, decreasing amount of microsilica the alkalinity reserve of the concrete is higher and the concrete cost is lower. Different additions have been used as replacement of Portland cement to reduce the carbon footprint and high costs of the UHPFRC. Yu et al. [17] assessed the influence of fly ash, slag and limestone powder. Lampropoulos et al. [18] developed concretes containing silica fume and slags. Pyo and Kim [19] pointed out that coal bottom ash, fly ash and slag powder can be effectively used. Lu et al. [20] used iron tailing powder and studied the mechanical performance and the microstructure under different curing conditions; they concluded that the steam curing regime was the most effective at early ages, while the warm-water curing had the best effect at late ages. Some authors also recommend the inclusion of nanoparticles (e.g., nanosilica, nano metaclay or calcium nanocarbonate) to enhance performance [21,22,23,24,25] or ground glass to lower costs [26,27,28]. These nanoparticles improve concrete properties due to their chemical reactivity and their physical effect (filler and nucleation effects). Nano metaclay delays the setting time, reduces the workability and increases the strength at late ages [25]. Nanosilica and nanocarbonate also reduce the workability but accelerate the hardening and increase the compressive strength at early age: nanosilica before 7 days and nanocarbonate mainly between 7 and 28 days [23].

In addition to a compressive strength of over 120–150 MPa, such concretes have a modulus of elasticity above 46 GPa, a tensile strength higher than 5 MPa and lower creep than conventional concrete [29]. Dynamic properties have also been studied, such as dynamic tensile strength under impact or fracture energy, although these parameters are strongly influenced by the experimental setup [16]. Adding steel fibres to the concrete mix improves its bearing capacity, ductility and shear strength [30,31], but the compressive strength is practically unchanged [32]. The fibre type has also an important influence on the mechanical properties. Tensile and bending strengths increase with an increase in the fibre dosage when hooked fibres are used. In the case of straight fibres, the strength does not rise at larger dosages. The increase in fracture energy is more pronounced with hooked fibres but a better spatial orientation of fibres is obtained with straight fibres [33]. An excessively high fibre content may induce clustering, but if air bubbles are produced, it may affect physical concrete properties, such as porosity and permeability [34,35]. These adverse effects can favour the penetration of aggressive agents and reduce the material’s durability.

To date, much research into this material has analysed its mechanical properties, and only a few studies on its microstructure or durability have been published. All the authors have observed lower porosity and water and gas permeability than in conventional concrete [36,37,38,39,40]. However, discrepancies have been identified in reported findings, due to their dependence on many factors, including type of additions and nanoparticles used [23,35,41] and curing conditions [42,43,44,45]. As the resulting UHPFRCs can vary in strength from 120 MPa to over 250 MPa, they are scarcely comparable. According to Pyo and Kim [19], porosity accessible to water ranges from 1% to 3% in UHPFRCs depending on steel fibre content, whereas Yu et al. [35] observed values of 10% to 17% in lower-strength UHPFRCs. According to some authors, water absorption is around 14-fold lower in UHPFRCs than in conventional concretes [36], while others report differences of up to 60-fold [37,38].

Concrete durability depends on its porous structure and, hence, its water and gas permeability. This is because most of the deterioration processes are linked to the aggressive agents transport through the concrete, and they modify its strength, stiffness and appearance. Therefore, it is important to know the microstructure and the transfer properties (porosity, permeability) in UHPFRCs. These properties are not yet well investigated in these concretes, and the few results available in literature are disparate. In addition, this research aims to add information of the response of UHs cured at air and high temperatures in contrast to the more typical standard curing at 20 °C and steam curing. Consequently, the objective of this experimental work is to study the porous structure of ultra-high performance concretes characterised by compressive strength at the low end of the range normally defined for this material, i.e., from 120 MPa to 150 MPa. In this way, the obtained characteristics can be deemed to correspond approximately to the minimum characteristics expected for this concrete type. The effect of a number of parameters, such as steel fibre content or curing conditions (air, 20 °C and high temperatures 60 °C and 90 °C), was analysed by comparing the results to the values observed in ordinary, high strength and very high strength concrete.

## 2. Materials and Methods

### 2.1. Concrete Mixtures and Materials

Six ultra-high-performance concretes (hereafter, UH) were made with three different fibre volume contents (0%, 1%, 2%) and four distinct curing conditions (laboratory environment, climate chamber at 20 °C, 60 °C or 90 °C). Conventional (C30), high-strength (C50) and very high-strength (C90) concretes were also made and cured for 28 days in a climate chamber at 20 °C and 95% RH. Three batches were prepared from each mix.

UHs were designed to the minimum compressive strength defined for such concretes when made without fibres and cured at 20 °C, i.e., from 120 MPa to 150 MPa. To simulate uncontrolled curing, the specimens cast with one of the UHs were allowed to be air-dried in the laboratory environment. The 60 °C and 90 °C concretes were stored at these temperatures and 100% RH for 48 h. They were subsequently cured at 20 °C and 95% RH through day 28, while the 20 °C concrete was cured under these conditions from the outset. The binder used in all four concretes was CEM I 42.5 R to which silica fume containing more than 90% silica was added. Two siliceous sand types, fine (0/0.5) and medium (0.6/1.2), were used as aggregate, together with silica flour with a similar particle size distribution to that of cement. Sika ViscoCrete 20 HE, the applied admixture, was batched to produce concretes with similar fluidity to that of self-compacting concrete. 

**Figure 1:**
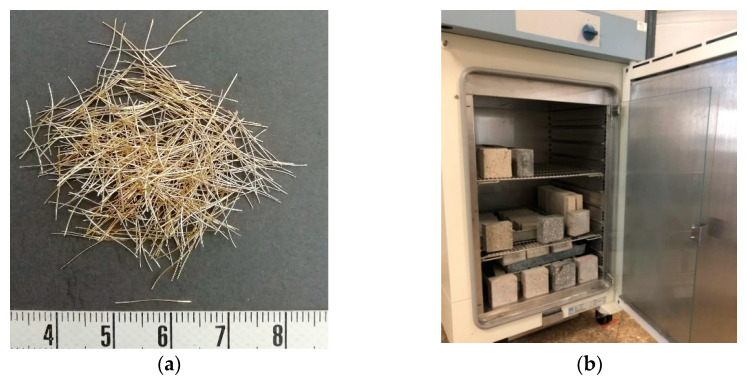
(**a**) Steel fibres used in the mixes; (**b**) Specimens in the climate chamber.

Short straight steel fibres 13 mm long and 0.2 mm in diameter, with an aspect ratio of 62 and a tensile strength of 2750 N/mm^2^, were also added to five of the six studied UHs (Figure 1). Fibres are a relevant parameter that influences the behaviour of UHs. These concretes have a great energy absorption capacity, but they own a brittle behaviour. They are also susceptible to autogenous shrinkage cracking due to the low w/c used [46]. Therefore, it is common to include fibres in the mixes to avoid a sudden failure, and control crack spacing and width, which in turns helps meet serviceability requirements. Some authors conclude that the addition of steel fibres decreases porosity [19], although other researchers point out an opposite effect [17]. Thus, the influence of this parameter on the porous structure of UHs should be considered. In this research, fibre content was limited to 2 vol.% because, according to Song et al. [47], higher contents do not significantly raise UHPFRC mechanical strength and may adversely affect durability. The experimental UHs were labelled to specify the proportion of added fibres (0F, 1F, 2F) and curing conditions (air, 20 °C, 60 °C, 90 °C).

Concretes C30, C50 and C90 were prepared with the aforementioned cement, and limestone aggregates consisting in 0/4 sand, 4/7 gravel and 4/12 gravel. The mix characteristics and respective compressive strength values are found in Table 1. The UH concrete with 2% of volume of fibres is a UH with a deflection-hardening behaviour, while the UH concrete with 1% of volume of fibres is a UH with a limit behaviour between deflection-hardening and -softening [48].

### 2.2. Test Methods

In order to analyse the porous structure of concretes, five tests were carried out: scanning electron microscopy (SEM), mercury intrusion porosimetry (MIP), thermogravimetry (TGA/DTG), water absorption and oxygen permeability. Compressive strength at 28 days was also determined from each mix in 100 mm diameter and 200 mm high cylindrical specimens (Table 1). Three batches were made from each mix and two samples were analysed per batch. The result of each mix was taken to be the arithmetic mean of the recorded six values. 

#### 2.2.1. Scanning Electron Microscopy (SEM)

At 28 days, samples were taken from inside the 100 mm cubic specimens to study concrete microstructure. Samples were carbon-coated to favour electrical conductivity. Materials were studied under a JEOL JSM6300 scanning electron microscope and a Zeiss Ultra 55 field emission scanning electron microscope fitted with an energy dispersive X-ray (EDX) spectrometer (SEMTech Solutions, North Billerica, MA, USA) for phase composition analyses.

#### 2.2.2. Thermogravimetric Analysis (TGA)

TGA was carried out with a Mettler TGA/SDTA 851e (Mettler Toledo, Barcelona, Spain) instrument to quantity the bound water and portlandite contents in concretes. The 50 mg samples taken from the 100 × 100 × 100 mm^3^ cubic specimens were crushed. Then the coarse aggregate was removed and samples were ground. Measurements were taken immediately after sampling. The material was tested at a heating rate of 10 °C/min from 25 °C to 1205 °C, under nitrogen flowing atmosphere at 75 mL/min.

#### 2.2.3. Mercury Intrusion Porosimetry (MIP) Test

At 28 days, pore size distribution was determined by a Micromeritics AutoPore IV-9500 mercury porosimeter (Micromeritcs GmbH, Mönchengladbach, Germany). The test was carried out on small drilled cores weighing approximately 6 g. The cored samples were obtained from the 100 × 100 × 100 mm^3^ cubic specimens. After oven-drying at 105 °C, samples were submerged in mercury and subjected to steadily increasing pressure. Total porosity was calculated by likening pore accesses to cylindrical capillaries whose radii, further to the Washburn-Laplace equation, were deemed inversely proportional to the applied pressure.

#### 2.2.4. Water Absorption Test

**Figure 2:**
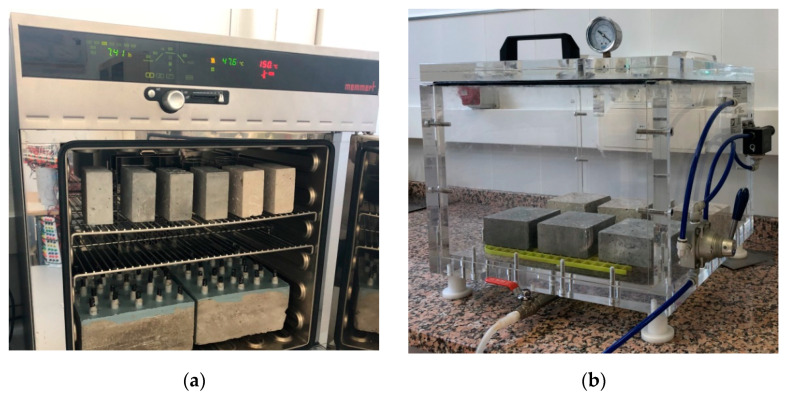
Water absorption set up: (**a**) specimens in the oven; (**b**) specimens in the vacuum chamber.

At 28 days, porosity accessible to water was found as specified in Spanish standard UNE 83980:2014 [49] on the 100 × 100 × 40 mm^3^ prismatic specimens oven-dried at 105 °C to constant weight (M_dry_). They were subsequently soaked in water for 72 h and then vacuum-dried in a chamber at a pressure lower than or equaling 20 mbar (Figure 2). Two hours later, the vacuum chamber was filled with water to a height of 20 mm on the specimens. After 24 h, specimens were removed from the chamber, and the hydrostatic (M_hyd_) and post-vacuum saturated (M_sat_) mass were found. These data were used to determine open porosity from the formula: 100 × (M_sat_ − M_dry_)/(M_sat_ − M_hyd_). Three batches were made from each mix and three samples were analysed per batch. The result of each mix was taken to be the arithmetic mean of the recorded nine values.

#### 2.2.5. Oxygen Permeability

**Figure 3:**
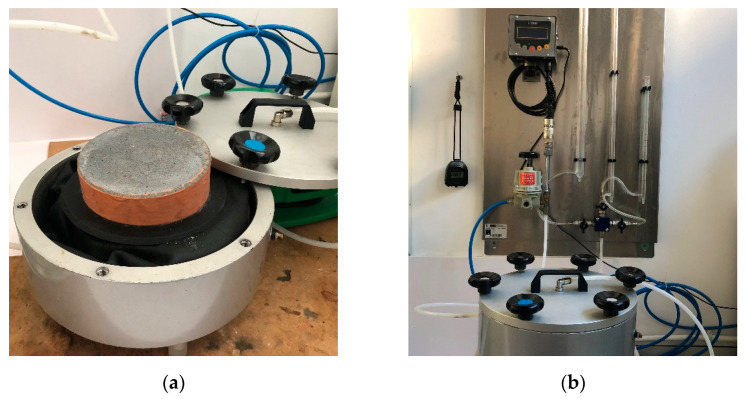
Gas permeability set up: (**a**) Specimen in the permeability cell; (**b**) Permeameter.

This method determines gas penetrability in hardened concrete by determining the gas permeability coefficient (K_p_), as recommended in Spanish standard UNE 83981:2008 [50], applicable to 28 days, in 15 cm diameter and 5 cm high cylindrical specimens. Specimens were epoxy resin-coated on their side surfaces prior to testing to prevent oxygen release. Tests were conducted in a permeability cell filled with synthetic air to eliminate the possible presence of water vapour, which would alter the findings (Figure 3). Permeability was measured at five air pressure settings in a Controls permeameter. Air flow was found by measuring the time it took a soap bubble to travel through a glass tube of known volume (in this case: 10 × 10^−6^ m^3^, 25 × 10^−6^ m^3^ or 100·× 10^−6^ m^3^). Three batches were made from each mix and three samples were analysed per batch. The result of each mix was taken to be the arithmetic mean of the recorded nine values.

## 3. Results 

### 3.1. Microstructure

#### 3.1.1. SEM Images

The morphology and the composition of the ultra-high performance concretes and the concretes C30, C50 and C90 have been studied. 

##### Morphology

**Figure 4:**
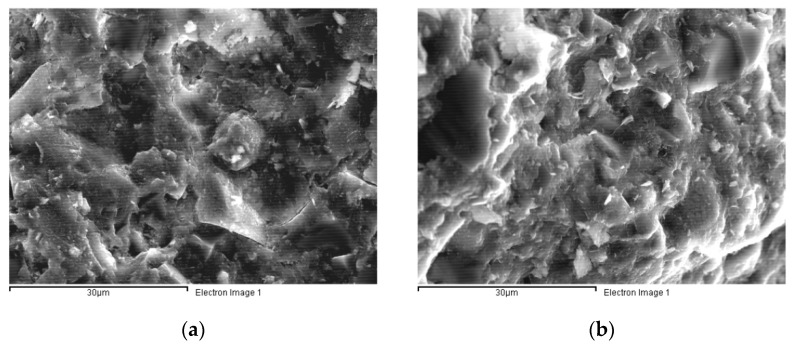
Micrographs (2000×) of concretes: (**a**) UH-2F-air; (**b**) UH-2F-90.

**Figure 5:**
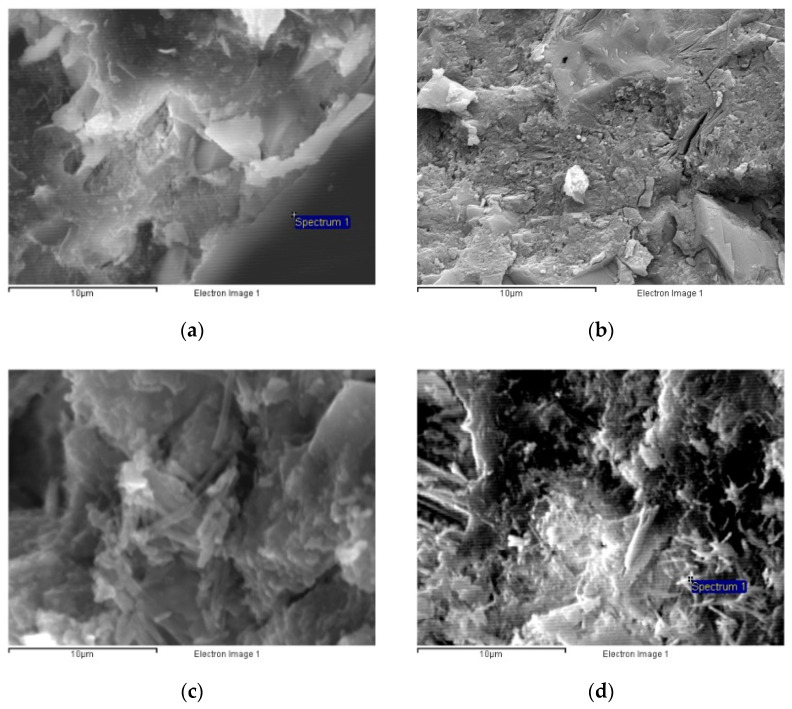
Micrographs (5000×) of concretes: (**a**) UH-0F-20; (**b**) C90; (**c**) C50; (**d**) C30.

All six studied UH types (with different fibre contents and cured under distinct conditions) exhibited very similar microstructures, with a very dense matrix (Figure 4). Whereas C90 was also characterised by a highly compacted cementitious matrix, in C30 and C50 voids were more numerous between the various phases in their cementitious matrices, and their structure was less homogeneous (Figure 5).

##### Composition

**Figure 6:**
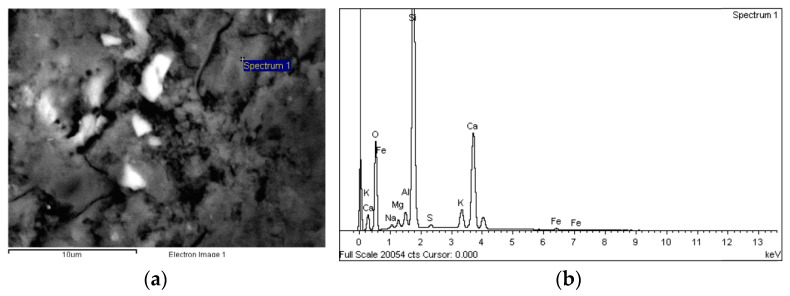
(**a**) BSE micrograph of UH-2F-60 (5000×) and (**b**) EDX spectrogram and chemical composition of the area identified on the micrograph.

The energy dispersive X-ray (EDX) analysis of the binder phases showed that the hydration products in UHs comprised essentially calcium silicate hydrates. These silicates, the grey areas in the back-scattered micrographs (Figure 6), were silica high in some zones, most likely due to the large amounts of silica fume added to the mix.

**Figure 7:**
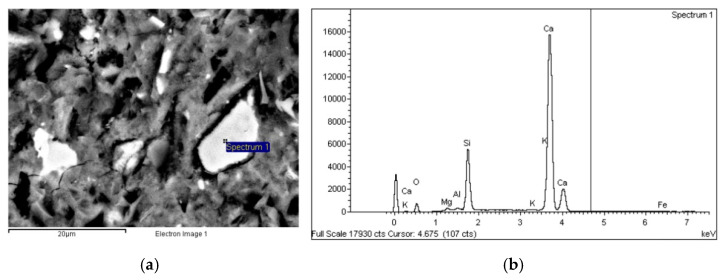
(**a**) BSE micrograph of UH-0F-20 (2500×) and (**b**) EDX spectrogram and chemical composition of the area identified on the micrograph.

**Figure 8:**
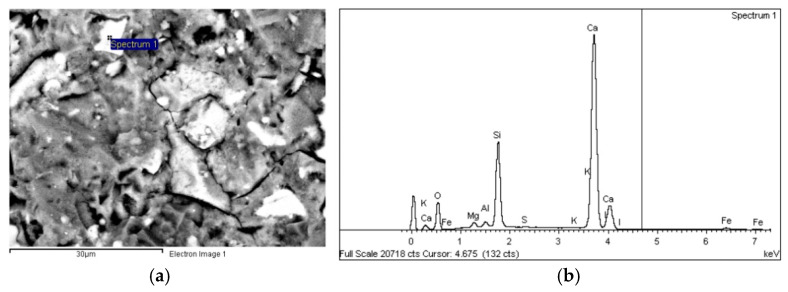
(**a**) BSE micrograph of UH-2F-air (2000×) and (**b**) EDX spectrogram and chemical composition of the area identified on the micrograph.

C_3_S and C_2_S, i.e., anhydrous cement grains, were also detected in significant amounts in the UHs (the whitish areas on the BSEM scans in Figure 7 and Figure 8). Moreover, no portlandite was detected in the analysed samples. This finding is consistent with earlier reports [3] and with the high silica fume content in concrete, which reacts pozzolanically with the Ca(OH)_2_ generated during cement hydration.

**Figure 9:**
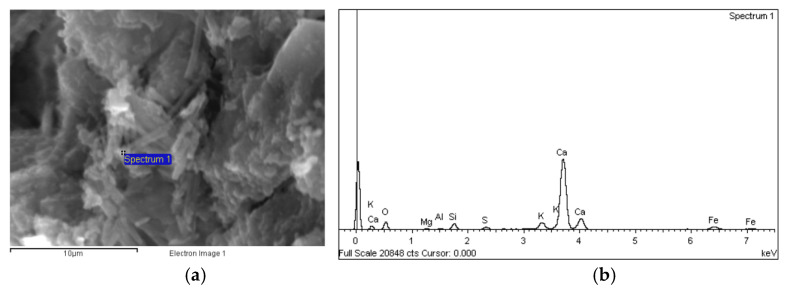
(**a**) BSE micrograph of C50 (5000×) and (**b**) EDX spectrogram and chemical composition of the area identified on the micrograph.

As regards concretes C30, C50 and C90, a number of crystalline structures and portland cement hydration products, such as calcium silicate hydrates and portlandite (Figure 9), were observed on the SEM micrographs. Although no ettringite crystals were identified in the analysed samples, the EDX analysis revealed Ca-Al-S phases, which suggests the presence of calcium sulfoaluminate hydrate (ettringite).

#### 3.1.2. Thermogravimetric Analysis (TGA)

**Figure 10:**
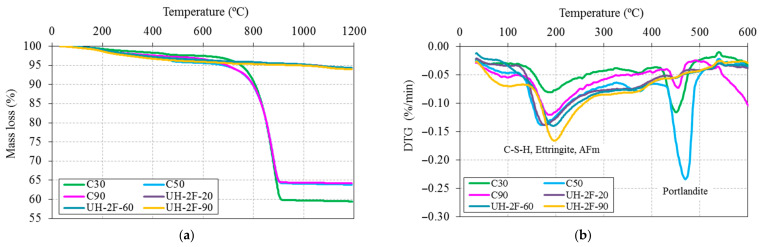
(**a**) TG; and (**b**) DTG curves of concretes.

The TG and derivative TG (DTG) curves determined by means of TGA are plotted in Figure 10.

The very significant (32% to 38%) mass loss recorded in concretes C30, C50 and C90 at 700 °C to 900 °C was attributable primarily to calcium carbonate decarbonation. These concretes contained large amounts of CaCO_3_, sourced from the limestone aggregate used in their manufacture, unlike the siliceous aggregates bearing UHs. Although tests were run up to 1200 °C, the curves plotted in Figure 10b show weight variation up to 600 °C to pay attention to loss of water due to the dehydration of hydration products (110 °C to 380 ºC) and calcium hydroxide dehydroxylation (380 °C to 520 ºC). Mass loss up to 380 °C is associated with the bound water [51] and is attributable to the dehydration of ettringite, C-S-H gel and AFm phases [52].

The bound water and portlandite contents estimated from the TGA findings are found in Table 2, where values are normalised to cement mass for inter-concrete comparison purposes. Portlandite content was calculated stoichiometrically from the following equation:

(1) $$CH=\;\frac{H}{m_{H}}m_{CH}$$

where *CH* is portlandite content, *H* is the water loss associated with portlandite decomposition, *m_H_* is molecular water mass and *m_CH_* is the portlandite molecular mass.

As shown in Table 2, UH concretes contained less bound water than concretes C50 or C90, which is consistent with the lower w/b ratio used and is indicative of a lower degree of cement hydration. Moreover, curing UH at temperatures of 60 °C and 90 °C induced a higher bound water content.

While portlandite was, in turn, detected on the DTG curves for all concretes (Figure 10b), it was practically negligible in UH concretes, a finding that is consistent with its absence in the SEM analyses. Specifically, Ca(OH)_2_ content was 30% lower in UH-2F-20 than in C90 and 75% lower than in C50 (Table 2).

#### 3.1.3. Pore Size Distribution

##### Effect of Concrete Type

**Figure 11:**
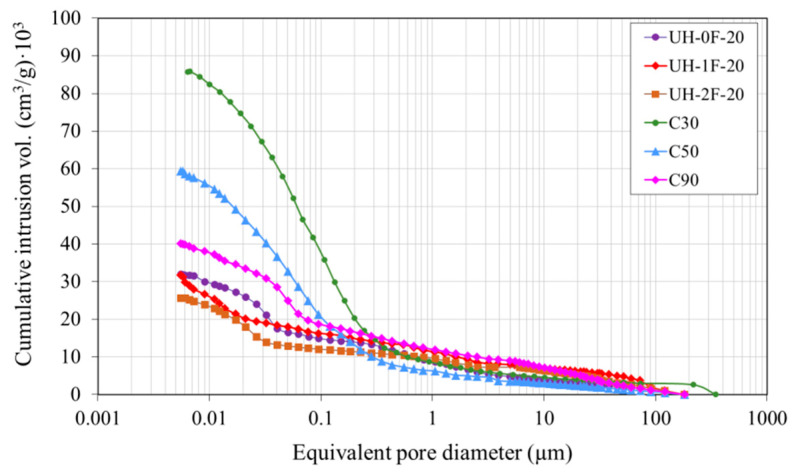
Cumulative pore size distribution.

**Figure 12:**
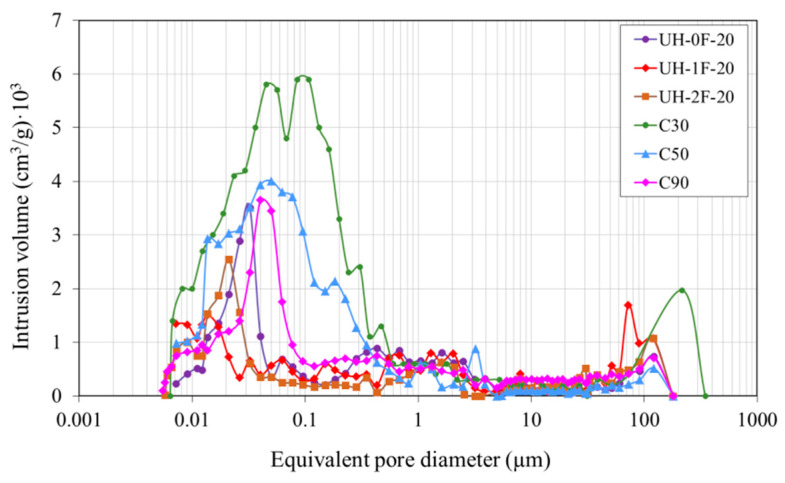
Pore size distribution.

Figure 11 and Figure 12 respectively show the mercury cumulative intrusion volume and mercury intrusion volume according to the equivalent pore diameter. Mixes differed most significantly in capillary pore volume (0.01 µm to 1 µm) which, together with macropore volume, affects material permeability.

The porosity in UH concretes was clearly lower than in concretes C30, C50 and C90 (Table 3). For instance, the total porosity in UH-0F-20 was 7.3%, while it was 17.6%, 13.1% and 10.0%, respectively, in C30, C50 and C90. Variation in fibre content (0%, 1% or 2%) did not affect the ultra-high performance concrete porous structure because both the total pore volume and pore size distribution were similar in UH-0F-20, UH-1F-20 and UH-2F-20.

Ultra-high-performance concretes had a higher porosity concentration on the nanoscale level (≤0.05 µm). In these concretes, the maximum pore concentration tended to be around smaller pore sizes, thus reducing the volume of larger capillary pores and increasing that of smaller capillary pores. For instance, in UHs the maximum pore concentration occurred for a pore size of 0.015–0.03 μm compared to 0.045 µm in C90, 0.05 µm in C50 and 0.10 µm in C30. 

Figure 12 also shows that the pore size, at which mercury intrusion rapidly increased, varied depending on concrete, and was much smaller in UHs. This pore size, labelled as the threshold diameter by some authors [53], defines the limit from which the biggest number of pores concentrate. Therefore, it offers a good indication of porous structure fineness. The threshold diameter is normally taken to be the smallest diameter exhibiting an intrusion volume differential of over 0.001 cm^3^/g [53]. Here the threshold diameter in UH-0F-20 was 0.05 µm, in C90 0.09 µm, in C50 0.40 µm, and approximately 0.60 µm in C30.

##### Effect of Curing Conditions on UHs

**Figure 13:**
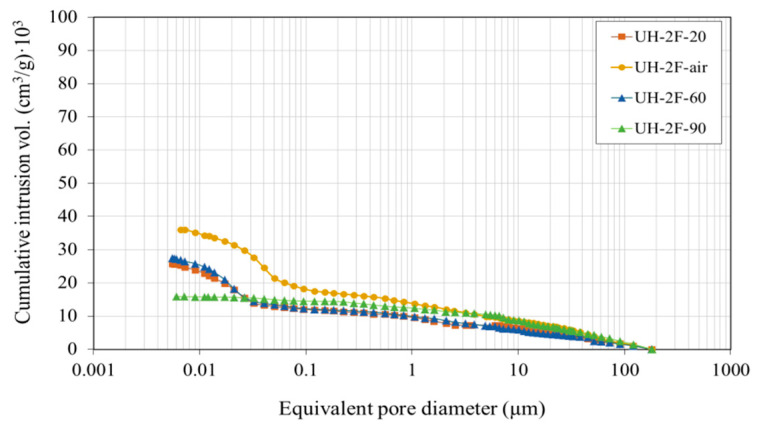
Cumulative pore size distribution.

**Figure 14:**
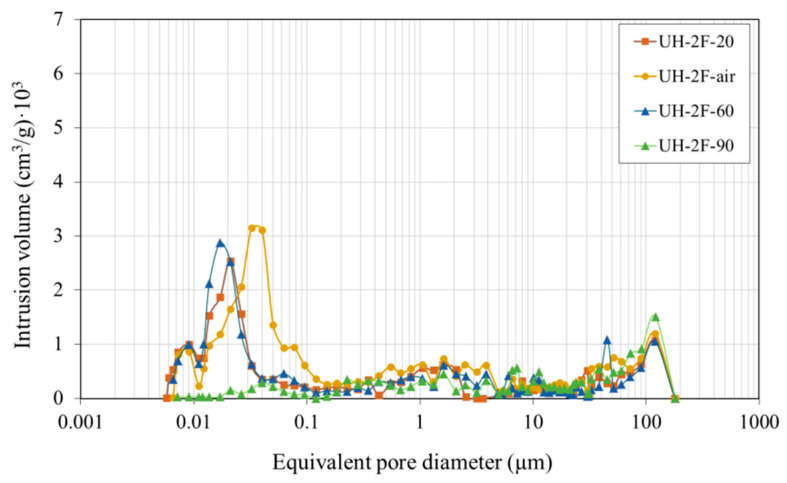
Pore size distribution.

A comparison was made of the concrete cured in a climate chamber at 20 °C and 95% RH (UH-2F-20) to the concrete cured in a laboratory atmosphere (UH-2F-air). It revealed a number of differences in both total pore volume (Figure 13) and porous structure (Figure 14). Total pore volume was, for instance, 50% bigger in UH-2F-air (Figure 13). Figure 14, in turn, shows that the porous structure was somewhat finer in UH-2F-20 than in UH-2F-air, in which the maximum pore concentration occurred for a pore size of 0.02 µm and 0.04 µm, respectively. Moreover, the threshold diameter below which the largest number of pores lay was 0.04 µm in UH-2F-20 and 0.15 µm in UH-2F-air. Therefore, the absence of controlled curing affected UH, although its total porosity was smaller than in concrete C90.

As shown in Figure 13 and Figure 14, only the highest curing temperature (90 °C: UH-2F-90) significantly influenced the porous structure. Although the findings were similar for the concretes cured at 20 °C and 60 °C (UH-2F-20 and UH-2F-60), the total pore volume was 40% lower in the specimens cured at 90 °C (Figure 13). Likewise, at 90 °C a notable decrease was noted in the pores smaller than 0.04 μm (Figure 14).

### 3.2. Porosity Accessible to Water (Water Absorption)

**Figure 15:**
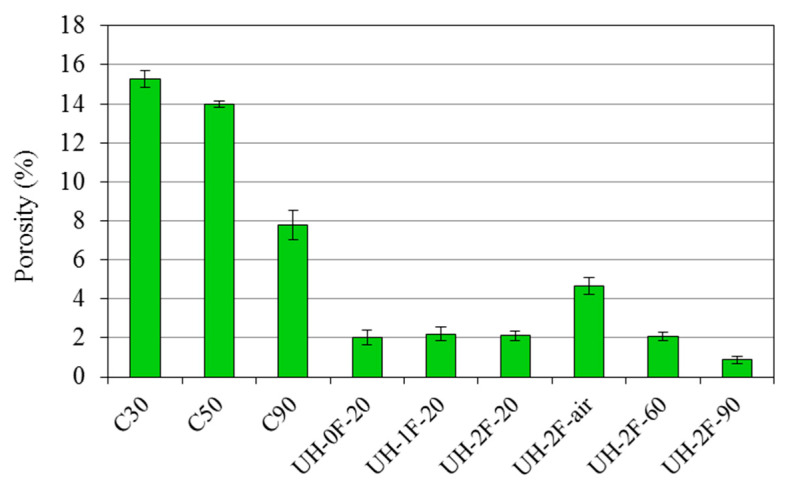
Porosity accessible to water.

As this parameter is closely related to concretes’ resistance to aggressive agent ingress, it provides an indirect durability measure. The porosity values based on the water absorption test at 28 days are shown in Figure 15.

#### 3.2.1. Effect of Concrete Type

The observed values lay within the ranges reported earlier by some authors [3]. As Figure 15 shows, at the age of 28 days UHs exhibited porosity accessible to water 7-fold lower than in C30, 6-fold than in C50 and 4-fold than in C90. According to the SEM and MIP analyses, this is attributable to a much denser cementitious matrix, a much smaller pore volume (particularly as regards large capillary pores, 0.05-1 µm) and a finer porous structure. 

For the influence of fibres on the water porosity of ultra-high performance concretes, no statistically significant differences were observed between concretes UH-0F-20, UH-1F-20 and UH-2F-20. That is, corroborating the MIP findings, the use of 0%, 1% or 2% fibre did not affect water permeability in these concretes.

#### 3.2.2. Effect of Curing Conditions on UHs

A comparison of the climate chamber-cured concrete (20 °C, 100% RH; UH-2F-20) to the concrete exposed to a laboratory environment (UH-2F-air) revealed significant differences, with porosity around 2-fold higher in the latter.

As observed in the MIP and TGA analyses, curing temperature was found to affect material properties, although the differences between 20 °C (UH-2F-20) and 60 °C (UH-2F-60) curing were not statistically significant. 

### 3.3. Oxygen Permeability

**Figure 16:**
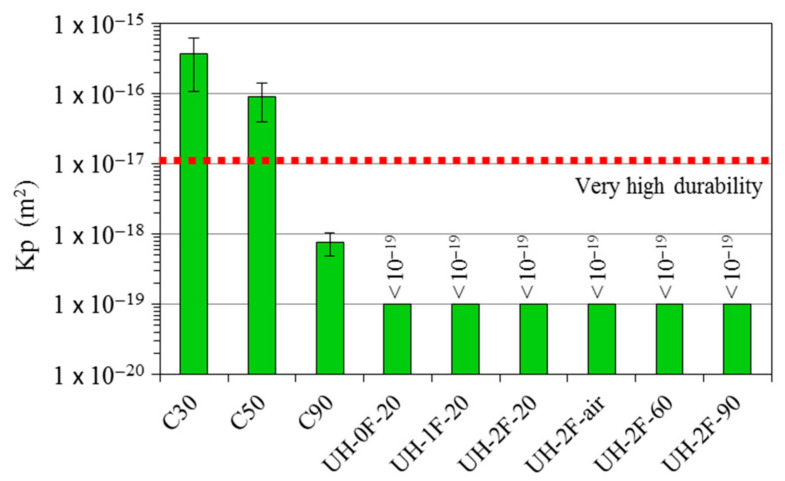
Oxygen permeability coefficient (K_p_).

Figure 16 shows the gas permeability coefficient (K_p_) values obtained from the gas permeability tests. This parameter is also a good indirect indicator of concrete durability, because it provides information on material porosity. Moreover, the reinforcement corrosion rate and onset time are associated with O_2_ and CO_2_ gas penetration across the concrete cover. As the K_p_ coefficient could not be measured in the ultra-high-performance concretes (at the highest pressure delivered by the facility, no air went through the specimen), the possible effect of fibre content or curing conditions and temperature on material permeability could not be analysed. The findings inferred that, given the applied pressure and specimen thickness, the K_p_ coefficient in these concretes was under 10^−19^ m^2^. Despite such measuring limitations, clear differences were observed between the ultra-high-performance concretes and concretes C30, C50 and C90, denoting much greater durability in the former, even for concrete UH-2F-air, which was cured under uncontrolled laboratory conditions. Oxygen permeability was, for instance, at least one order of magnitude lower in concrete UH-2F-20 than in C90, two orders of magnitude lower than in C50 and three orders of magnitude lower than in C30.

## 4. Discussion

### 4.1. Effect of Concrete Type

SEM imaging reveals certain morphological and compositional differences between the ultra-high performance (UH) and the other analysed concretes. The high compacity of UH explains their high mechanical strength (128 MPa to 150 MPa at 28 days). With respect to the UH composition obtained with EDX analysis, the high amount of anhydrous cement grains indicates that cement hydration is incomplete. This incomplete hydration is caused by its low w/b ratio (0.19), typical for these concrete types, which denotes that the amount of water is not enough to react with all the binder.

The UHs analysed are characterised by a very low portlandite content, nearly 30% lower than in concrete C90 and 75% than in C50. The lower portlandite content in the UHs is due to the low w/b ratio, but also to the calcium hydroxide consumption during the pozzolanic reaction between silica fume and portlandite. Some authors also point out that portlandite content decreases as the w/b ratio lowers because the transport of involved ions in the formation of new hydrates is hindered by the paucity liquid phase [54]. 

From the durability point of view, a decrease in the Ca(OH)_2_ content is not desirable, since it produces a subsequent decrease in the pH value, which can alter the stability of the passive oxide layer that protects rebars, leading inevitably to corrosion onset. However, the results of SEM analysis indicate that UHs contain large amounts of anhydrous cement particles, and thus, they have a large alkaline reserve and a latent Ca(OH)_2_ reserve which can be generated in the presence of water. Furthermore, UHs highly dense microstructure produces high resistivity matrices due to their low porosity (total porosity around 30%, 45% and 60% lower than in concretes C90, C50 and C30, respectively). Due to their low porosity, the dissolved ions cannot circulate easily through the pore solution, and thus, these matrices have extremely reduced ionic current. This property adds an additional intrinsic protection from corrosion when using UHs, which is an extra benefit from other concrete types.

In addition, the porous structure of UHs is very fine, with a high concentration of pores on the nanoscale level, which also justifies the excellent durability performance of this family of concretes, since it is difficult for water and gases to penetrate the gel pores. The highest fineness of the porous structure of UHs, particularly compared to C30 and C50, is due to a number of factors, including: (1) the higher coarse aggregate content in concretes C30, C50 and C90 as porosity is higher and capillary pore size is larger in the aggregate-paste interfacial transition zone (ITZ) than in cement paste [55]; (2) the higher fines content in UHs, which have a filler effect and constitute nucleation sites favouring greater and more effective cement hydration; (3) the silica fume added to UHs and C90, since due to the pozzolanic reaction with portlandite, porosity decreases because ITZ thickness is reduced as a result of the depletion of the store of portlandite crystals [56]; and (4) the use of a more powerful superplasticizer in UH, C90 and C50 than in C30, which favours cement dispersion and, hence, reduces the formation of flocs inside which pores form [57].

As result of these microstructural characteristics, the transport properties differ greatly in the concrete types studied in this research. These differences can be seen directly through porosity accessible to water and oxygen permeability, which are indirect indicators of the material durability. The porosity accessible to water in UHs at 28 days is 7-fold lower than in C30, 6-fold than in C50 and 4-fold than in C90. The oxygen permeability is three orders of magnitude lower than in C30, two orders of magnitude lower than in C50 and one order of magnitude lower than in C90. This result adds relevant information on the potential durability of a structure made with the different concrete qualities.

### 4.2. Effect of Fibre Content

The volumetric content of fibres in UH (with 0%, 1% or 2%) does not affect the total pore volume, the pore size distribution, nor the water permeability. This result contrasts to other research published in which the content of fibres was reported to be influential due to the potentially larger ITZs [17]. This suggests that an appropriate particle size distribution of the mix and a homogeneous distribution of the fibres during the mixing may be enough to ensure that the presence of the fibres does not increase the porosity of the UHs. In fact, in this study, UHs with 1% and 2% of volume of fibres have comparable total porosity, pore size distribution and porosity accessible to water to that of the UH mix without fibres. Fibres also have other beneficial impacts that could help to counteract possible adverse effects on the durability due to weak interfaces (higher porosity at the steel-paste interface), that are able to improve the durability of an element at the structural level. One of these major contributions is that fibres contribute to control shrinkage cracking, which can be significant in concretes with a high cement content, such as in the case of UHs [58,59]. In this regard, fibres can prevent the development of cracks and hence water seepage is reduced.

### 4.3. Effect of Curing Conditions

The curing conditions in UHs affect greatly their microstructure. At 90 °C there is a 40% decrease in total pore volume and the porous structure is much finer, giving rise to a denser cementitious matrix. In addition to expediting hydration reactions, a temperature of 90 °C induces higher water vapour pressure, favouring easier and faster gas penetration through the porous network and with it both cement hydration and the silica fume pozzolanic reaction. Likewise, curing UH at temperatures of 60 °C and 90 °C induces a higher bound water content and, consequently, greater cement hydration, which would explain the higher compressive strength in concretes UH-2F-60 and UH-2F-90 than in UH-2F-20. The portlandite formation is also influenced by the curing temperature. The Ca(OH)_2_ content in the UH cured at 60 °C and 90 °C is 14% higher than in the UH cured at 20 °C. This fact is an outcome of the higher degree of cement hydration in the concretes cured at higher temperatures.

As result of a higher cement hydration, a smaller pore volume and a finer porous structure, concrete cured at 90 °C is substantially less permeable to water than concrete cured at 20 °C, being the porosity accessible to water around half (0.9% compared to 2.1% at the age of 28 days).

The lack of curing in UH also generates an 50% increase in the total porosity and a coarser porous structure, giving rise to a porosity accessible to water 2-fold higher than in UH cured in a climate chamber at 20 °C and 95% RH. However, concrete compressive strength was not affected by lack of curing (Table 1), which suggest the idea that porosity increased only on the surface of specimens, which is the zone most strongly affected by an uncontrolled curing. Despite this higher permeability, the porosity accessible to water recorded in concrete UH-2F-air (around 4.7% on average) lie within the range defined in the French design guide [3] for ultra-high performance concretes (1.5% to 5%), and clearly below the values proposed by Baroghel-Bouny [60] for very high durability concretes (6% to 9%). Moreover, even when cured in ambient conditions, UH is less porous than very high strength concrete C90, in which the mean 28-day value was 7.8%.

## 5. Conclusions

The conclusions drawn from the analyses conducted to determine the microstructure and permeability of the UHs prepared with different steel fibre contents and under distinct curing conditions and temperatures are set out below. UHs were designed to the minimum compressive strength defined for such concretes when made without fibres and were cured at 20 °C, i.e., from 120 MPa to 150 MPa. In this way, the obtained performance can be deemed to correspond approximately to the minimum performance expected for this concrete types.

The analysed UHs are characterised by a very low portlandite content, nearly 30% lower than in concrete C90 and 75% lower than in C50. However, they contain large amounts of anhydrous cement grains, which provides them a large alkaline reserve and, consequently, a good protection against carbonation-induced corrosion. UHs have also a very dense microstructure, with a total porosity 30%, 45% and 60% lower than in concretes C90, C50 and C30, respectively. This low porosity adds an additional protection from corrosion because pore connectivity diminishes and, therefore, the ionic current through the dissolved ions in the pore solution is hindered. In addition, the porous structure of UH is very fine, with a high concentration of pores on the nanoscale level, which also justifies the excellent durability performance of these concretes, since it is difficult for water and gases to penetrate the gel pores.

As result of these microstructural characteristics, the transport properties differ greatly in the concrete types studied in this work. The porosity accessible to water in UHs at 28 days is 7-fold lower than in C30, 6-fold than in C50 and 4-fold than in C90, and the oxygen permeability is three orders of magnitude lower than in C30, two orders of magnitude lower than in C50 and one order of magnitude lower than in C90. This result adds relevant information on the potential durability of a structure made with the different concrete qualities.

The use of different fibres contents in UH (0%, 1% or 2%) does not affect the total pore volume, the pore size distribution, nor the water permeability. This result contrasts to other research published in which the content of fibres was reported to be influential due to the potentially larger ITZs. This suggests that an appropriate particle size distribution of the mix and a homogeneous distribution of the fibres during the mixing may be enough to ensure that the presence of the fibres does not increase the porosity of the UHs. 

The curing conditions in UHs affect greatly their microstructure and consequently their permeability. The degree of cement hydration rises with curing temperature (20 °C to 60 °C to 90 °C), inducing a rise in portlandite content. The total pore volume is around 40% lower in UH cured at 90 °C than in UH cured at 20 °C. Likewise, the porosity accessible to water is around half the value observed for the 20 °C concrete. On the other hand, the lack of curing in UH generates an 50% increase in the total porosity and a coarser porous structure, giving rise to a porosity accessible to water 2-fold higher than in UH cured in a climate chamber at 20 °C and 95% RH. Notwithstanding, the water porosity is lower than in concrete C90.

## Figures and Tables

**Table 1:** Mixture proportions of concretes (kg/m^3^) and compressive strength (MPa).

	UH-0F-20	UH-1F-20	UH-2F-20	UH-2F-air	UH-2F-60	UH-2F-90	C30	C50	C90
Cement	800	800	800	800	800	800	300	450	500
Water	160	160	160	160	160	160	192	225	178
Superplasticizer	30	30	30	30	30	30	2.8	1.37	3.5
Silica fume	175	175	175	175	175	175	---	---	55
Silica flour	225	225	225	225	225	225	---	---	---
Sand (0/0.5)	302	302	302	302	302	302	---	---	---
Sand (0.6/1.2)	565	565	565	565	565	565	---	---	---
Sand (0/4)	---	---	---	---	---	---	1256	880	914
Gravel (4/7)	---	---	---	---	---	---	---	880	779
Gravel (4/12)	---	---	---	---	---	---	707	---	---
Steel fibers	---	80	160	160	160	160	---	---	---
w/b ^(*)^	0.19	0.19	0.19	0.19	0.19	0.19	0.65	0.50	0.33
f_c_ (28 days)	129.8 (3.71)	129.9 (3.58)	128.4 (5.88)	137.9 (7.26)	145.7 (6.10)	154.2 (5.99)	31.6 (4.40)	49.9 (3.89)	88.9 (5.78)

(*) includes the aqueous part of the superplasticizer; Numbers in brackets are the standard deviation (MPa).

**Table 2:** TGA-determined bound water and portlandite contents (cement wt%).

	C30	C50	C90	UH-2F-20	UH-2F-60	UH-2F-90
Bound water (110–380 °C)	0.47	0.58	0.38	0.29	0.30	0.34
Portlandite (380–520 °C)	1.09	1.37	0.49	0.36	0.41	0.41

**Table 3:** MIP results.

	UH-0F-20	UH-1F-20	UH-2F-20	UH-2F-air	UH-2F-60	UH-2F-90	C30	C50	C90
Volume of pores (cm^3^/g)	0.032	0.032	0.026	0.039	0.028	0.016	0.086	0.059	0.041
Porosity (%)	7.3	7.6	6.7	9.6	6.8	4.0	17.6	13.1	10.0
Threshold diameter (μm)	0.05	0.03	0.04	0.15	0.04	--	0.60	0.40	0.09

## Data Availability

The data presented in this study are available on request from the corresponding author. The data are not publicly available due to further research.
